# Meanings of participation in care for older people after hip fracture surgery and nurses working in an orthopaedic ward

**DOI:** 10.1080/17482631.2021.1970302

**Published:** 2021-08-25

**Authors:** Cecilia Segevall, Kerstin Björkman Randström, Siv Söderberg

**Affiliations:** Department of Nursing Sciences, Mid Sweden University, Östersund, Sweden

**Keywords:** Hip fracture, narrative interviews, nurses, older people, participation, phenomenological hermeneutics

## Abstract

**Purpose:**

The aim of this study was to elucidate meanings of participation in care for older people after hip fracture surgery and nurses working in an orthopaedic ward.

**Methods:**

A qualitative phenomenological hermeneutical design was used. We conducted personal interviews with a narrative approach with 11 older people recovering from hip fracture surgery and 12 nurses working in an orthopaedic ward.

**Results:**

The results show that for older people, participation meant being a co-creator in their own care, founded on being met with sensitivity and support, being told what is going to happen, taking responsibility and asking questions and being able to influence care. For nurses, patient participation meant meeting the patients’ needs and requests by being open and allowing them to influence care while at the same time recognizing that the patients’ possibility to influence care was limited.

**Conclusion:**

The study shows that for older people and nurses, the phenomenon of participation has similar meanings but also differences. When older people participate in their care, they become a co-creator in care and confirmed as a person. This highlights the importance of a nurse-patient relationship built on trust, connectedness and communication based on a shared understanding.

## Background

Patient participation in care has been discussed for decades due to its importance to healthcare and patients. Efforts have been made worldwide to strengthen patients’ opportunities to participate in and make decisions regarding their care (World Health Organization, 2017). For older people, participating in care and decision making during a hospital stay strengthens their abilities to manage daily life post-discharge (Lyttle & Ryan, 2010). Furthermore, older people have an increased risk of suffering a hip fracture, a condition that represents a serious threat to their health (Parker & Johansen, 2006) and rapidly changes their daily life (Bruun-Olsen et al., 2018; Rasmussen & Uhrenfeldt, 2016). For many older people, hip fractures are a trauma that creates a sense of losing control over their lives (McMillan et al., 2012; Segevall et al., 2019) as well as affects the individual physically, mentally and socially for a long time (Peeters et al., 2016). It takes time to adjust to not being able to live life the way the person used to prior to the hip fracture, and dependence upon family and friends is huge (Segevall et al., 2019). In this situation, it would seem to be of the utmost importance that these older people are offered the opportunity to influence and participate in their care.

Participation is a broad concept that is interchangeably used with involvement, engagement and collaboration (Snyder & Engström, 2016). The concept is used in different contexts and has been described from the perspectives of patients, their relatives as well as healthcare professionals (Dyrstad et al., 2015; Tobiano et al., 2015). Patient participation can be defined as: “A patient´s rights and opportunities to influence and engage in decision making about his care through a dialogue attuned to his preferences, potential and a combination of his experiential and the professional’s expert knowledge” (Castro et al., 2016, p. 1929). Patient participation is proposed to be dependent on an established relationship; a surrendering of some power or control by the nurse; shared information and knowledge; and an active mutual engagement in intellectual and/or physical activities (Sahlsten et al., 2008). The concept relates to and includes learning, caring relationships and reciprocity (M. Nilsson et al., 2019).

Patient participation has been described as beneficial for patients. Patients who participate in their own care are, for instance, more likely to be satisfied with the care they receive when compared to patients who do not participate (Weingart et al., 2011). The feeling of being involved can provide a sense of security and control of the future (Alharbi et al., 2014), which, in turn, may affect the outcome of care due to enhanced concordance with treatment and motivation to improve health. It may also lead to a sense of independence and wellbeing in terms of enhanced confidence, reduced anxiety and better relationships with the healthcare professionals (Lyttle & Ryan, 2010).

There are several challenges surrounding the realization of patient participation (Tobiano et al., 2015). Patients’ preferences for participation ranges from not wanting to participate at all to wanting to make autonomous decisions. The preference for participation can vary within the same patient depending on time and context (Thompson, 2007). Other patients think that they are not competent enough and, thus, trust the medically trained healthcare professionals to make decisions in their best interest (Alharbi et al., 2014; Larsson et al., 2011). In addition, some patients lack information to make decisions and end up agreeing to suggestions from the healthcare professionals (Segevall et al., 2018).

A nurse’s manner challenges participation. Nurses who use condescending or de-personalized language or choose to not verbally engage leave the patient feeling ignored (Tobiano et al., 2015). Likewise, an attitude characterized by power and control, in which the nurse does not pay attention to the patient’s opinions or wishes, hinders participation (Larsson et al., 2011). Time constraints and heavy workloads challenge patient participation in such a way that healthcare professionals tend to keep information to a minimum, leaving patients uninformed (Dyrstad et al., 2015). As conditions and challenges for patient participation are closely intertwined, in the absence of factors that enable participation, the patient is left uninvolved. Conditions that enable participation occur when the patient is informed according to his/her needs in order to make independent decisions and respected for his/her knowledge (Eldh et al., 2006).

Research about patients’ experience of participation in care during a hospital stay has shown that patients do not always feel involved in care nor are they given the opportunity to make their own decisions (Bagnasco et al., 2019; Dyrstad et al., 2015; Malmgren et al., 2014). Information has been suggested to be a key aspect; to be involved in decision making, one has to be sufficiently informed about, for instance, the procedures they are going to undergo as well as their care plan (Bagnasco et al., 2019; Segevall et al., 2018). Å. Nilsson et al. (2018) found that being prepared for the hospital stay is critical for experiencing participation in care. A study by Florin et al. (2006), who compared registered nurses and patients’ preferences for participating in decision making, showed that patients prefer to take a relatively passive role in decision making during a hospital stay, while the nurses tend to overestimate the patients’ willingness to assume a more active role.

Several studies (Alharbi et al., 2014; Florin et al., 2006; Malmgren et al., 2014; Å. Nilsson et al., 2018; Tobiano et al., 2015) have explored the concept of patient participation in nursing care, but few have addressed it from the perspectives of both older people and nurses. To the best of our knowledge, this is the first study that has addressed the phenomenon of participation from two different perspectives in the specific orthopaedic context. Understanding meanings of participation for older people and nurses in an equal context allows an increased possibility for older people to be involved in their own care and participate based on their preferences, needs and capacities. Thus, the aim of this study was to elucidate meanings of participation in care for older people after hip fracture surgery and nurses working in an orthopaedic ward.

## Materials and methods

### Design

This study has a life world perspective reffering to the world we live in together with others and do not reflect upon i.e., the world we take for granted. The life world takes into account humans´own experiences and understandings of themselves, their bodies, and the meaning their life situations hold for them (Dahlberg et al., 2008).

A phenomenological hermeneutic method inspired by Ricoeur (1976) was used to elucidate meanings of participation for older people recovering from hip fracture surgery and nurses working in an orthopaedic ward. The method has been developed for nursing research by Lindseth and Norberg (2004) and is a research method for elucidating people’s lived experiences of a phenomenon. The method builds upon the beliefs of the French philosopher Paul Ricoeur, who linked the life world theory of phenomenology to an hermeneutical focus on interpretation and understanding (Lindseth & Norberg, 2004).

### Context

The study was conducted in a region of Mid-Sweden at a hospital that supplies health care to the entire county. In the orthopaedic ward investigated, nearly 300 patients undergo hip fracture surgery every year, and most of them are older than 65 years of age. The average length of hospital stay is about one week. Nursing care for older patients with a hip fracture focuses on maximizing mobility and preserving optimal function and involves monitoring signs that could interfere with the patinets capability for early mobilization such as signs of anaemia, pain, or poor nutritional status (Maher et al., 2012, 2013). The healthcare professionals in the orthopaedic ward works according to the Swedish hip fracture care programme which involves a short amount of time between admission to the hospital and surgery and early mobilization. They also work according to the Patient Act legislation which promotes patient participation in decisions about care (SFS, 2014:821).

### Participants and procedure

A purposive sample of 11 older people (6 women and 5 men) and 12 registered nurses (all women) participated in the study. The participants were recruited from an orthopaedic ward, where the older people had been admitted with a hip fracture and the nurses worked. The head of the orthopaedic unit consented to help with the recruitment of older people and let the nurses participate during working hours.The number of participants in the study was guided by the concept of information power, which emphases sample suitability and data quality to provide nuanced and rich descriptions (Malterud, Siersma & Guassora, 2016).

The inclusion criteria for the older people were to be a Swedish-speaking man or woman, 65 years of age or older and admitted to the orthopaedic ward with a hip fracture. They had to be oriented to their person, time, space and situation, and willing to talk about their experience. The participants were informed about the study by the discharge planning nurse during their hospital stay. Those who were interested left their name and phone number for the research team to contact them once home. The first author contacted the participants to further inform them about the study and ask if they were willing to participate. If so, a date and place for the interview were scheduled. Sixteen older people agreed to be contacted by the research team, and 11 chose to participate. The older people were between 65 and 84 years old (Md = 75). Four participants lived alone, and seven lived with a spouse.

The inclusion criteria for the nurses were to be Swedish speaking and to have worked in the orthopaedic ward for at least six months as a nurse. The nurses were informed about the study via their workplace email by the first author and those interested in participating were asked to respond to the email. The first author contacted interested nurses to set a date and place for the interview. Twentyfive nurses met the inclusion criteria, 12 chose to participate. The nurses were between 26 and 63 years old (Md = 42) and had worked at the orthopaedic ward between eight months and 19 years (Md = 5.5 years). Five of them had a master’s degree in nursing science.

### Interviews

Personal interviews with a narrative approach (Mishler, 1986) were conducted with the participants. Narratives are stories collected from individuals about their lived experiences. The stories may talk about the individuals’ past, present and future (Creswell, 2013; Mishler, 1986). The interviews were conducted one-on-one by the first author, between June 2019 and April 2020. The interviewer is a doctoral student and registered nurse with extensive experience with working with patients admitted to the orthopeaedic ward. The interviewer was not involved in care of the older people in this study. Data were obtained using interview guides on the older people’s and the nurses’ lived experiences of participation in hospital care after hip fracture surgery. The interview guides were based on prior research. Before the beginning of each interview, the first author repeated the information about the study in order for the participants to ask questions regarding their participation in the study.

The interview guide for the older people consisted of three questions (see Table I). Follow-up questions like “Can you give an example?” “Can you tell me more about that?” and “How did that make you feel?” were asked when clarification was needed. The interviews took place 1–4 weeks after discharge. Seven interviews took place in the participant’s home, and four interviews were conducted over the phone. The interviews lasted between 30 and 86 minutes (Md = 44) and were audio recorded and transcribed verbatim.

**Table I.** Interview guide for the older people

1. Please tell about your hospital stay with a focus on participation in care. 2. Please tell about a situation in which you felt you participated in care. 3. Please tell about a situation in which you did not feel you participated in care.

The interview guide for the nurses consisted of three questions (see Table II). Follow-up questions like, “Can you give an example?” “Can you tell me more about that” and “How did that make you feel?” were asked when clarification was needed. The interviews took place in a separate room at the nurses’ workplace, lasted between 38–92 minutes (Md = 42) and were audio recorded and transcribed verbatim.

**Table II.** Interview guide for the nurses

1. Please tell about how you work with older patients admitted with a hip fracture at this orthopaedic ward. 2. Please tell about situations in which the patient participates in care. 3. Please tell about situations in which the patient does not participate in care.

### Analysis—The phenomenological hermeneutic interpretation

The verbatim transcribed interviews were analysed with phenomenological hermeneutic interpretation. This research method consists of three interrelated phases: naïve reading, structural analysis and comprehensive understanding. In the first phase, naïve reading, the interview texts were read several times to get a grasp of their immediate meaning as a whole. In the next phase, structural analysis, the texts were divided into meaning units, condensed and reflected upon regarding similarities and differences. The condensed meaning units were then grouped based on similarities into subthemes and themes. In the final step, comprehensive understanding, the preunderstanding of the authors, naïve understanding and results of the structural analysis were reflected upon in relation to the aim and the context of the study and relevant literature to reach a comprehensive understanding of the participants’ lived experiences of the phenomenon of interest (cf. Lindseth & Norberg, 2004). According to Ricoeur (1976), there are several ways to interpret a text, but not all interpretations are equally probable. The interpretation presented in this study is the one that we found to be the most probable. To enhance rigour, all authors were involved in the analysis, and different interpretations were discussed until an understanding was reached.

#### Ethical considerations

The study was conducted in accordance with the declaration of Helsinki (World Medical Association, 2018) and approved by an Ethical Review board. Participants received oral and written information about the study before giving their voluntary informed consent to participate. The information included the aim of the study as well as the participant’s right to withdraw from the study at any time without giving a reason. The participants were guaranteed confidentiality and assured that the findings would be presented without mentioning names or other identifying information.

## Results

### Naïve understanding

#### Older people

It seemed as if participation in care was important to some extent. This meant that things would work out well when returning home since being involved in care and planning for discharge. Information and communication in dialogue were a part of participation. Participation meant being informed about what was going to happen as well as why, so prepartion for what to come was possible and gave opportunities to seek support from relatives when needed. It seemed difficult to influence care, since the healthcare professionals followed a strict schedule based on carrying out activities on routine. Older people seemed lacking expectations in influencing or making decisions regarding care and trusted that healthcare professionals knew what they were doing. The only thing older people seemed to be a part of was planning for discharge, since having concerns about how things would work out at home. As older people wanted to be seen and listened to, a condition for participation was that healthcare professionals appearing to be nice and encouraging. Participation meant taking a more active role and asking questions.

#### Nurses

It appeared to be difficult to express what participation meant, although it was important for the outcome of care. It seemed that patients were not often asked for their suggestions and had little or no opportunity to influence care; however, in some situations, the patient could participate and choose. Making patients participate was depending on the patients themselves and time. A condition for participation was that the patient did not suffer from dementia or mental illness, since the patient then was considered not to understand their own needs. If the work situation was too stressfull it meant that the possibility of participation was reduced. Participation meant listening to and asking the patient questions as well as taking a step back and trusting the patient to make good decisions. It seemed that when opinions differed from the patient’s opinion, nurses tried to convince the patient to change his/her mind. Participation meant information and communication in dialogue.

### Structural analysis

The analysis revealed one theme including four subthemes for the older people (Table III) and three themes including seven subthemes for the nurses (Table IV). In the subsequent section, the theme with subthemes for the older people are presented first, followed by the themes and subthemes for the nurses, illustrated with quotations from the interview texts.

**Table III.** Overview of the theme and subthemes of the interview texts of the older people (n = 11)

Theme	Subtheme
Being a co-creator in their own care	Being met with sensitivity and support Being told what is going to happen Taking responsibility and asking questions Being able to influence

**Table IV.** Overview of themes and subthemes of the interview texts of the nurses (n = 12)

Theme	Subtheme
Meeting needs and requests	Being able to rethink according to the patient’s wishes Informing and educating the patient Respecting the patient’s will
Giving possibilities to impact	Seeing the patient as part of the team Letting the patient be in control
Limiting the patient’s involvement	Permitting the patient to influence Depending on the patient

## Older people

### Being a co-creator in their own care

The theme Being a co-creator in their own care includes the following subthemes: Being met with sensitivity and support; Being told what is going to happen; Taking responsibility and asking questions and Being able to influence.

#### Being met with sensitivity and support

Nice and welcoming healthcare professionals made older people feel included and participative. It was important that healthcare professionals was caring and that they were able to laugh together. Feeling that the healthcare professionals seemed interested in them as people, asking how they were doing was considered valuable. Participation meant being met with sensitivity and an ability to adjust activities according to the older people’s capacities. Situations where it was unable to perform activities according to the healthcare professionals instructions and when no explanation was given gave feelings of disappointment, which was followed by a lack of participation in care.

I used that thing that was hanging above my bed [overhead trapeze] to get out of bed, but she [the staff] wanted me to push out of bed using my arms instead. I told her my arms weren´t strong enough, but she said there was no other way.

Participation could mean being met with patience acknowledging that the staff had to deal with patients that all seemed to need a great deal of help and support. Although that had to be tiresome, the healthcare professionals never seemed to show a sour face. Healthcare profesionals sometimes made demands that seemed impossible to older people like getting out of bed on the first day after surgery but encouring words facilited making it possible. This seemed to give feelings of participation. Important for feeling paritcipative was when healthcare professionals saw improvements since the last time they saw each other.

When they [the staff] came back after their day off, they noticed that I had made improvements, and they let me know it. That really made me feel strengthened.

Older people felt seen and supported when healthcare professionals sensed that they were upset or in distress, asking if there was something the staff could do to make them feel better. Having supportive relatives was a help to being participative and meant feeling fortunate to have relatives that were willing to give support. Older people would inform their relatives about what was happening on the ward and what the doctor said during rounds. When concerns about how things would work out after discharge, older people discussed the matter with their relatives. Relatives suggested refurnishing to have easy access to the bed to lie down and rest during the day, and offered to help with household tasks which led to older people not feeling the need to have municipal social services at discharge.

I asked my husband what he thought, and he said that we would manage on our own, he´d help me with what I needed. So I declined municipal social service.

Older people being met with sensitivity and support meant that they both receiving what they needed at the hospital and felt assured that things would be as expected when returning home.

#### Being told what is going to happen

Being in a hospital environment could be experienced as alien, especially for older people who never been admitted to a hospital before. The need for someone to informing about routines and what to expect was great and considered to be part of participation. Healthcare professionals in general were informative and kept older people up to date about things that were going to happen. This meant that older people could begin to mentally prepare as well as inform their relatives. Healthcare professionals guided them through procedures by letting older people know what they were doing and why, which was educational and reassuring.

They told me lots of things and explained that we have to do this now and so on, so I felt that I knew what was happening.

Older people also experienced situations when they were not informed in accordance with their needs. In these situations, older people felt they were treated differently than the others, which made them feel left out; this was seen as the opposite of participation.

When they came to take blood pressures in the morning, they would take it on the others but not me. I don´t know why, but I didn´t exactly feel wanted.

#### Taking responsibility and asking questions

Older people saw it as their responsibility to ask questions to stay informed and participative. Instead of waiting for information from the healthcare professionals, older people took matters into their own hands. Older people engaged in dialogue with healthcare professionals and felt free to let the staff know what they wanted and how they wanted things to be carried out if they had specific requests. Although trusting healthcare professionals, older people made sure the staff were aware of their medical history, so that nothing would be missed.

After surgery, I needed a blood transfusion. I was a bit anxious because I have antibodies, so I told them that to make sure they were aware of that.

Asking questions about things that were unclear was a presumption for participation. Healthcare professionals would sometimes forget to answer questions and older people had to ask several times before receiving an answer. Getting answers was important for participation, even though they also accepted that all questions could not be answered.

Early Saturday morning, they came to draw blood and I kept asking what the result was and was I to be discharged that day? They said they would check, but it wasn´t until 4 PM that they told me the doctor wanted me to stay overnight.

#### Being able to influence

The feeling of having the opportunity to influence care strengthened older people’s self-esteem, which was pivotal to recovery, health and participation. Desiring to influence was not large and older people felt that requests had been accommodated, such as choosing what to eat for dinner and receiving pain medication when needed; these actions were of importance for feeling participative. Older people who wanted to be discharged earlier than planned were allowed to do so. It was difficult to influence daily care, since healthcare professionals seemed to follow a strict schedule where activities were performed on routine. Nevertheless, older people were content with that and did not feel the need to influence or make decisions about care.

No, I didn´t feel that I could influence what happened at the ward, it was just to string along. But that was okay with me.

Important for participation was being involved in the decision regarding municipal home care services. Older people desired to decide whether to employ these services and what kind of help could be suitable for their needs. Situations where older people were not able to influence this decision was in contrast to participation. Instead, older people were told during rounds that they were going to be discharged later that day. As a result, older people had to stay at the hospital for a couple more days to make the proper arrangements. This situation meant that older people did not participate in their care.

I hadn´t heard a word about it [discharge planning with social worker from the municipal home care services], and I would have wanted to know because then I could have told them that I couldn´t go home and manage by myself.

## Nurses

### Meeting needs and requests

The theme Meeting needs and requests includes the following subthemes: Being able to rethink according to the patient’s wishes; Informing and educating the patient and Respecting the patient’s will.

#### Being able to rethink according to the patient’s wishes

Based on previous experiences, nurses knew what the patient was going to need during the hospital stay and after discharge by only talking to the patient and reading his/her journal at admission. While these actions were deemed as important to make the patient feel involved in his/her care, the patient sometimes had a different idea. To accommodate patients’ requests, nurses had to be flexible and able to rethink and adjust original plans. This could mean postponing physical activity with the physiotherapist due to pain or not feeling well. When planning for discharge, this could mean initiating or not initiating discharge planning with the social worker in the municipality. Nurses strived to rethink according to the patient’s wishes, but, due to lack of time and a busy workload in the ward, this was not always possible. This meant that patients who wished to stay an extra day were not allowed to do so. Those situations affected nurses emotionally, since it meant that all patients did not have the same opportunities. The experience is that care was not equal and in contrast to participation.

Sometimes, it´s not possible to accommodate everyone’s needs the way that they want to be accommodated. In those situations, there´s not so much participation to speak of. Instead, we have to tell the patient no.

#### Informing and educating the patient

Informing the patient about what was going to happen and explaining why was pivotal for participation. Nurses acknowledged that patients were in a vulnerable position due to the hip fracture. Providing information at an early stage meant that patients could start to prepare by thinking about their needs after discharge, which was a prerequisite for participation. To make sure that the patient had understood, nurses gave the same information several times and invited the older people to ask questions.

I tell them what is going to happen and why I do certain things. I encourage them to let me know if there is anything I can do, whatever that might be.

Meeting the patients’ needs meant educating in self-care, such as how and when to take their medication as well as monitoring signs of wound infection. When it was busy at the ward, patients did not receive information as normal, nor get their questions answered to the same extent. This situation influenced the patients opportunity to be involved in care.

#### Respecting the patient’s will

Meeting the patient’s wishes and needs meant respecting what the patient wanted, which was of importance for making the patient feel participative. When nurses’ and the patient’s beliefs about what was best differed, nurses tried to convince the patient to change his/her mind by informing them about the negative consequences of such a decision. Sometimes nurses got patients to picture themselves managing on their own at home as a way of trying to change their mind. Respecting the patient’s decision could mean a risk for post-operative complications, a readmission solely with the need for discharge planning with the municipality, or, at worst, a new fracture; this was ethically challenging for the nurses.

I can feel a certain amount of frustration because, I mean, I have tried to help with pain relief, and they keep saying no. I can see that they are in pain, but it is still their choice to make.

### Giving possibilities to impact

The theme Giving possibilities to impact includes the following subthemes: Seeing the patient as a part of the team and Letting the patient influence and be in control.

#### Seeing the patient as a part of the team

It was important to see the patient as a part of the team as the patient contributed with knowledge about his/her needs and resources, and the healthcare professionals contributed with medical and nursing expertise. Seeing the patient as part of the team was important for participation, as this meant taking advantage of and assembling all the factors that could increase the patient’s independence during and after the hospital stay. The patients had become more involved in care during recent years, but there was still room for improvement. It was natural for the patient to participate in care by saying yes or no to things as long as they understood to what they were saying yes or no. Nurses experienced difficulties explaining what participation meant, it was something involving more than solely making decisions. Nurses rarely or never reflected upon the meaning of it—but were convinced practicing patient participation regardless.

I´ll have to think for a while [silence]. For me, patient participation means that I get to say what I think … and it´s about what I can influence … but I also think about information – when you receive information, you´re participating.

#### Letting the patient influence and be in control

An involved patient was more motivated to mobilize and adhered to what was going on, which made nurses’ work easier. This meant that everyone was striving towards the same goal—that when the patient leaves the hospital, he/she is as independent as possible. As a result the patient could be discharged from the hospital earlier than expected. By making the patients participate, nurses believed that they felt safe at discharge and would manage better at home since they had been involved in planning and, thus, would receive the help they needed. Patients who participated in care left the hospital satisfied and with a positive attitude towards care.

If you have been listened to and had the opportunity to say what’s on your mind, I believe that you experience the hospital stay in a positive way.”

Letting the patient influence care was related to taking the time to listen and get to know the patient as a person. It meant creating a dialogue where the patients could share feelings and thoughts, followed by the nurse’s reply to their concerns. Nurses tried to establish trust to reassure patients who were feeling anxious about going home. By letting the patient influence care, nurses had to take a step back and let the patient take the lead. This meant not being in total control over the situation, which nurses could find difficult depending on the situation as well as the patient.

### Limiting the patient’s involvement

The theme Limiting the patient’s involvement includes the following subthemes: Permitting the patient to have influence and Depending on the patient.

#### Permitting the patient to have influence

Influencing care was limited to decisions in daily care after the hip fracture surgery. Prior to surgery, the patient’s opportunity to choose and make decisions was very restrained, since the patient lacked the competence to decide on medical issues. Participation in this stage was related only in terms of keeping the patient informed. The patient’s opportunity to influence was also limited after surgery due to routine procedures, such as checking vital signs, drawing blood and post-operative x-ray. Patients seldom questioned such procedures and seemed content by having them done. When the patient had undergone surgery, nurses meant that patients had influence over everyday activities, such as deciding when to get up in the morning and choosing what to eat from a menu.

It´s important that patients participate, but it´s also important that they get care that is of high quality. And for that to happen, you might have to structure it a bit.

#### Depending on the patient

The level of participation in care depends on the patient. Patients who were younger and cognitively alert were more participative, since they were competent in taking a stand and making decisions that would be good for them. For patients who were cognitively impaired, participation seemed to be a paradox, as they could influence care by terminating an intravenous line by removing it, but had no influence when it came to decisions on everyday activities or discharge planning. For these patients, participation was made possible by contacting relatives who made decisions on their behalf. Relatives became involved if the patient suffered from mental illness or requested assistance because he/she felt unwell. When there were no relatives available, healthcare profesesionals made decisions for the patient according to their beliefs of what the patient might have wanted.

Many patients suffer from some sort of cognitive failure, and those have no [silence] they can influence here and now, but they are not involved in discharge planning.

### Comprehensive understanding and reflections

Comprehensive understanding, the last phase in the interpretation process, contains a reflective reading of the text as a whole, taking into account the authors’ preunderstanding, the naïve understanding and the results from the structural analysis. To understand meanings of participation in care among older people admitted with a hip fracture and nurses working at an orthopaedic ward, the theory of caring and uncaring by Halldorsdottir (1996) and prior research were used. The themes and subthemes that elucidate participants’ lived experiences of participation show similarities as well as differences, reflecting various dimensions of meanings of the phenomenon. From the perspective of older people, the phenomenon of participation can be understood as being a co-creator in their own care; this is founded on being met with sensitivity and support, being told what is going to happen, taking responsibility and asking questions as well as being able to influence care. From the nurses’ perspective, the phenomenon of patient participation can be understood as meeting the patients’ needs and requests by being open to and allowing them to influence care while also recognizing that the patients’ possibility to influence care was limited.

For older people participation in care meant being met by sensitive and kind healthcare professionals who made them feel welcomed and cared for. Healthcare professionals that seemed interested in them as people when the staff asked them how they were feeling seems to be an expression of a caring relationship. A caring relationship is characterized by mutual trust, and a connectedness between the patient and the nurse and is founded on mutual respect, kindness and open communication. A caring relationship is symbolized by a bridge between the nurse and the patient (Halldorsdottir, 1996). From this, it can be understood that the older people felt connected to the healthcare professionals and confirmed as a person, which seems to be important in patient participation, i.e., a demonstration of the bridge between the patient and the nurses. Confirmation, which can be described as having one’s feelings acknowledged by an important other, produces feelings of hope, comfort and confidence and is an important part of recovery. Further, confirmation can be seen as a result of when patients perceive healthcare professionals as warm and nurturing (Drew, 1986). In light of Buber, 2008 thoughts on ‘I–It’ and ‘I-You’ relationships, this can be understood as the older people feeling that they were seen as a person—a subject, rather than a patient—object. According to Buber, 2008, an ‘I-You’ relationship is the basis for human fulfilment, and it is through these connections that human beings grow and develop. The nurses in this study took the time to listen to the patient and showed interest in the patient as a person and this is essential in making the patient participate in care. This can be understood as the nurses reaching out to their patients by showing interest in them; according to Halldorsdottir (2008), this is the beginning of a development of a caring connection where the nurses are building a bridge between themselves and the patient in order to meet the patient’s needs and requests.

The participants in this study experienced that information was an important part of participation in care. The older people needed someone to inform about routines and what to expect. For them, this provided a sense of security and helped them to prepare for things to come. This can seen as older people becoming more vulnerable and uncomfortable in their new situation when admitted to the hospital. Patients who are admitted to the hospital may experience existential changes involving the lived experience of uncertainties, vulnerabilities, and discomfort (Halldorsdottir, 1996). The vulnerability that affects patients results in the dignity of that person being threatened (Gastmans, 2013), but when patients are informed according to their needs and have knowledge about what to expect, they become less vulnerable (Irurita, 1999). For the nurses in this study, giving information meant providing knowledge and guidance through the new situation of being a patient. We interpreted this as the nurses acknowledging older people’s need for guidance and informing them in accordance with their needs, which helps the older people become less vulnerable while protecting their dignity. This can be seen as an expression of what Halldorsdottir (1996) described as a bridge in the relationship between nurses and patients.

Older people experienced that participation in care also meant taking an active role and asking questions when they wanted to know something; they also engaged in dialogue with the healthcare professionals. This can be understood as the older people feeling that they were in a safe environment and that asking questions would not be seen as questioning or distrusting the healthcare professionals. When patients feel safe to ask questions and raise concerns about care, their dignity is protected (Kerr et al., 2020). For the older people in this study, taking responsibility and asking questions can be understood as them feeling comfortable with the health care personnel. When patients are met with sensitivity and compassion, a relationship built on trust and understanding emerges in which an honest mutual sharing of information in both directions is made possible (Pentecost et al., 2020), i.e., a communication based on a shared understanding. When patients take the initiative to ask questions, they tend to ask about things that are of importance to them, which may not necessarily be the same as the nurses (Kettunen et al., 2002). For the older people this was a way to gain knowledge that will support their participation in care from their perspective.

For older people, being able to influence daily care was a part of participation. They felt that in situations where they had made requests, they had been listened to and, when possible, accommodated. However, they also experienced difficulties in influencing daily care, since healthcare professionals performed activities by routine; nevertheless, they were satisfied with the care received and seldom questioned it. This can be seen as if being listened to and feeling like someone important, is more worthwhile than actually making decisions. Everyone wishes to be confirmed and valued for what they are and what they can become (Buber, 1957). The nurses in this study experienced that the patients’ opportunities to influence care were limited when admitted with a hip fracture due to the nature of the hip fracture care programme as well as the patients’ lack of medical competence; however, the patients were informed about the procedure and care, and their opportunities to participate increased after the hip fracture surgery. In contrast to older people, the nurses experienced that the patient influenced all parts of daily care, as they never forced someone to do something he/she did not want to do. The different experiences described by older people and nurses can be understood as a way for the nurses to be in control over care and by their actions, the nurses do not invite the patient to participate in daily care. Nurses prefer routines rather than incorporating the patient’s preferences into care, since that lets them maintain control over care, thus impeding participation (Tobiano et al., 2016). This is in line with what Halldorsdottir (1996) calls a wall, which can be seen as the result of a disconnection between the nurse and the patient. The wall occurs when the nurse is perceived as inattentive, lacking in genuine concern and interest for the patient or as being indifferent to the patient as a person.

Older people experienced situations where they did not feel participative, such as not having their needs of support accommodated and not being informed according to their needs. These situations made the patient feel unimportant. According to Halldorsdottir (1996), how patients perceive the encounter is based on their lived experience and involves their needs, expectations and previous experience. In our study, older people feeling unimportant can be understood as a violation of a person’s dignity, which is the opposite of confirmation. This seems to be an important aspect to consider, since it can affect the patients’ health not only during their current hospital stay but also during their future encounters with healthcare.

Nurses in this study found it difficult to explain what participation in care meant, as they had never thought about its meaning before. They were convinced that it was important for outcome of care. It seems as the nurses see participation as something they do on an everyday basis and take for granted; therefore, they may not realize that it is not taken for granted by the patient. Although nurses described participation in terms that are in line with current definitions, it is still accompanied by some confusion, which shows the complexity of the phenomenon of patient participation. Raising awareness of meanings of patient participation can help nurses change how they view their interactions with patients (Tutton, 2005) and can be seen as a means to tear down the wall between the patient and the nurse, and enhancing possibilities for the patient to participate in care.

## Methodological considerations

The participants in this study were chosen by purposive sampling. Purposive sampling means that the participants were selected to best meet the informational needs of the study. The major criticism of this type of sampling is that the sample is biased by the selection process, meaning that a certain type of participants with a certain type of knowledge are encouraged to participate in the study. However, this criticism does not take into account that this is the focus in using this method (cf. Morse, 1991). Purposive sampling is a tool to provide a theoretical richness in seeking to elucidate the experiences as richly and accurately as possible. Interviews with a narrative approach were used, and the older people as well as the nurses spoke freely about their experience of patient participation in care and gave rich narrations. The fact that the last four interviews with the older people were conducted over the phone can be seen as a limitation. However, this was a result of the recommendations on social distancing due to the COVID-19 outbreak. While these interviews were shorter and, they were just as filled of depth and rich descriptions as those that were conducted face to face.

In qualitative research, the researchers’ preunderstanding is pivotal. According to Lindseth and Norberg (2004), the meaning of a phenomenon disappears without preunderstanding, and, for that reason, it cannot be put aside in brackets. In this study, we were aware of our preunderstanding, and the interpretation has been performed from the perspective of our experiences and understanding of patient participation in healthcare.

## Conclusions

In this study, being a co-creator in own care, being listened to and confirmed seems to be a prerequisite for participation in care for older people after hip fracture surgery. Nurses need to be aware that a nurse-patient relationship built on trust and connectedness in which the nurse is genuinely interested in the patient promotes participation. The nurse-patient relationship does not happen by chance, it needs to be created with care, skills and trust. This study indicates that meanings of patient participation have both similarities and differences for older people and nurses in an orthopaedic context. Following Halldorsdottir (1996), there seems to be either a bridge or a wall in the communication between older people and nurses, which influences the opportunities for older people to be participative in their care. To strengthen patient participation, nurses could use discussion groups to raise their awareness on the phenomenon of patient participation. They need to be aware and strive for communication based on a shared understanding. Thereby, nurses build bridges and avoid walls in communication with the patient. This is important to take in consideration in planning care for older people after hip fracture surgery in order to make participation possible.
